# Infliximab therapy for Crohn’s-like disease in common variable immunodeficiency complicated by massive intestinal hemorrhage: a case report

**DOI:** 10.1186/1756-0500-7-382

**Published:** 2014-06-22

**Authors:** Yuko Akazawa, Fuminao Takeshima, Hiroyuki Yajima, Daisuke Imanishi, Tsutomu Kanda, Kayoko Matsushima, Hitomi Minami, Naoyuki Yamaguchi, Ken Ohnita, Hajime Isomoto, Tomayoshi Hayashi, Masahiro Nakashima, Kazuhiko Nakao

**Affiliations:** 1 Department of Gastroenterology and Hepatology, Nagasaki University Hospital, 1-7-1 Sakamoto, 852-8501 Nagasaki, Japan; 2 Department of Tumor and Diagnostic Pathology, Atomic Bomb Disease Institute, Nagasaki University Graduate School of Biomedical Sciences, Nagasaki, Japan; 3 Department of Haematology, Nagasaki University Hospital, Nagasaki, Japan; 4 Department of Pathology, Nagasaki University Hospital, Nagasaki, Japan

**Keywords:** Common variable immunodeficiency, Inflammatory bowel disease, T lymphocyte, Colitis

## Abstract

**Background:**

Common variable immune deficiency is the most frequently encountered immunodeficiency in adults, which is characterized by low levels of serum immunoglobulins. Common variable immune deficiency can present with inflammatory bowel disease-like colitis because of the dysregulated immune system; paradoxically activated T cell receptor pathways are thought to be pivotal in pathogenesis of common variable immune deficiency-related colitis. Treatment for severe complications, such as gastrointestinal bleeding, is not established. We report a case of common variable immune deficiency-related Crohn’s-like disease presenting massive melena, which was successfully treated by short course infliximab therapy.

**Case presentation:**

A 26-year-old Japanese man with history of common variable immune deficiency presented with diarrhea, abdominal pain, and fever. Venous administration of antibiotics did not improve his symptoms. Colonoscopy revealed multiple longitudinal ulcers as well as cobblestone-like change in the ileum end and the ascending colon. Histopathological examination of biopsy specimen showed erosion and infiltration of T lymphocytes with lack of B cells. Intravenous hyperalimentation, mesalazine, and steroid did not improve the symptoms and the patient subsequently presented with massive melena. Colonoscopy revealed a protuberant vessel on one of the ulcers in the ascending colon. Endoscopic clipping was repeatedly performed for hemostasis, which was only temporarily successful. In an attempt to manage the bleeding and colitis, a trial of infliximab was given on week 0, week 2 and week 6. Gastrointestinal hemorrhage from the ulcer halted immediately after the first infliximab injection. Colonoscopy performed after the third infliximab showed remarkable improvement in the ileocolitis. No evidence of increased susceptibility to infections was observed and the patient has been in clinical remission for 3 years.

**Conclusions:**

We present this case together with review of literature to share our experience of encountering common variable immune deficiency complicating severe Crohn’s-like disease and to support that infliximab is a safe and effective treatment that can promptly manage life-threatening intestinal hemorrhage in common variable immune deficiency-related colitis.

## Background

Common variable immunodeficiency (CVID) is one of the most frequent symptomatic primary immunodeficiency encountered in adults, and is characterized by low level of immunoglobulin (Ig) G, IgM, IgA and lack of B lymphocytes, and some T-cell defects [1-3]. Delay in diagnosis and treatment of CVID can lead to serious infections and possible death. The incidence of CVID is considered to be from 1:50,000 to 200,000 [4]. CVID usually has a complex and heterogeneous genetic basis, and the known monogenic CVID defects can explain only a small group of patients [5].

Since gut is the largest lymphoid organ in the body which contains the majority of lymphocytes and producing immunoglobulin, malfunction of the regulatory mechanisms maintaining the balance between active immunity and tolerance in the gastrointestinal tract can lead to mucosal inflammation. Indeed, gastrointestinal disorders are relatively common manifestations and often the initial presenting symptom in patients with immune dysfunctions and are observed up to 60% of the patients with primary immunodeficiency including CVID [2]. Nevertheless, incidence of inflammatory bowel disease (IBD)-like colitis is reported to be as low as 2–4% in these patients [6]. Tumor necrosis factor alpha (TNF-α) inhibitors including infliximab have shown significant effects in classic IBD, while there is a concern that these treatments can be accompanied by an increased vulnerability to infections in CVID patients [3,7]. Although beneficial use of anti-TNF alpha agents has been reported in a few patients with Crohn’s-like disease in immunodeficiency to date [3,6], it is unclear whether they can also improve acute complications such as gastrointestinal hemorrhage. Here, we report a case of Crohn’s-like disease in CVID leading to challenging intestinal bleeding, which was successfully controlled by infliximab.

## Case presentation

**Figure 1:**
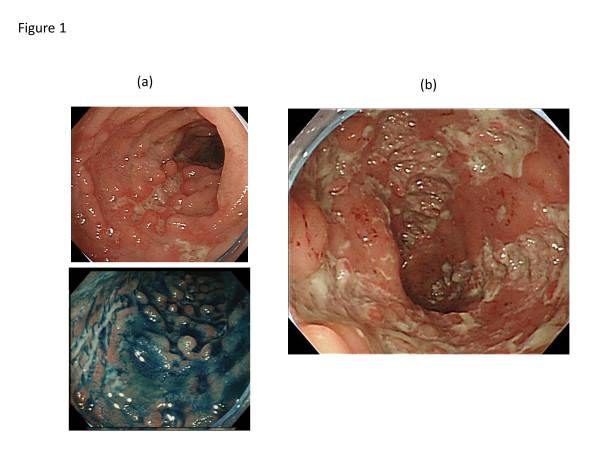
**Colonoscopy images of Crohn’s-like disease.** Colonoscopy on onset of diarrhea demonstrating multiple longitudinal ulcers and Cobblestone change of the mucosa in the ileum end **(a)** and deep ulcerations in the ascending colon **(b)**.

**Figure 2:**
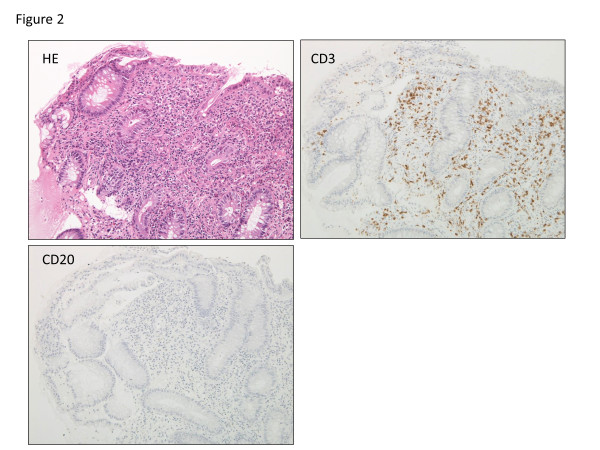
**Pathological images of Crohn’s-like disease.** Pathological examination of the ascending colon showing crypt distortion, infiltration of inflammatory cells including T cells, and lack of B cells in the mucosa.

**Figure 3:**
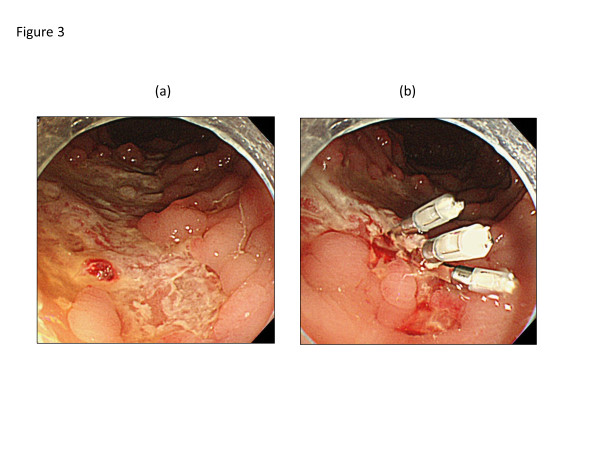
**Colonoscopic clipping of an exposed blood vessel.** Emergency colonoscopy showing an exposed blood vessel on the edge of a liner ulcer in the ascending colon **(a)**. Clipping was performed **(b)**.

**Figure 4:**
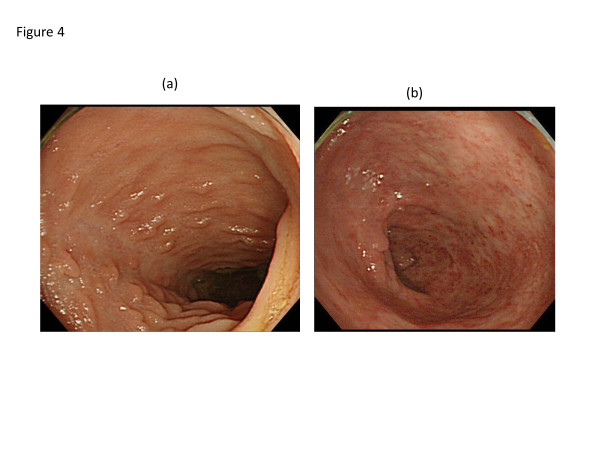
**Marked improvement of Crohn’s-like disease after infliximab therapy.** Follow-up colonoscopy showing healing of ulcers in the ileum end **(a)** as well as marked improvement on lesions in the ascending colon **(b)**.

**Figure 5:**
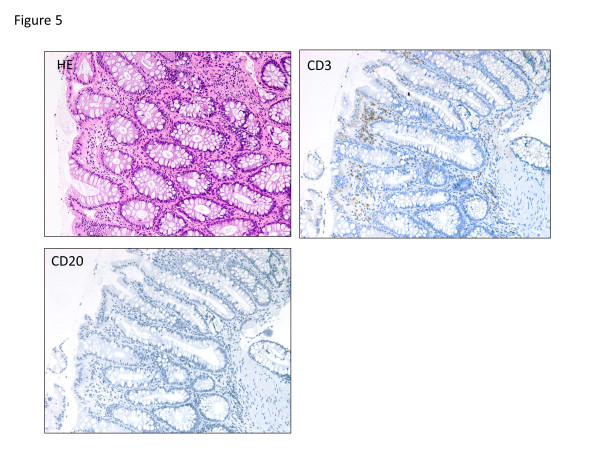
**Improvement of pathological findings after infliximab therapy.** Pathological examination of the ascending colon showing mucosal healing and reduced infiltration of CD3+ T cells.

A 26-year-old Japanese man complaining of diarrhea, abdominal pain, and fever, was transferred to our hospital after treatment with oral antibiotics had failed. Three years prior to this admission, the patient was diagnosed with CVID and had been receiving monthly intravenous immunoglobulin infusions. He also had a history of treatment for tuberculosis and repeated pneumonia. The patient had not been on non-steroidal anti-inflammatory drugs (NSAIDs), proton pump inhibitor (PPI), or other medications that can lead to intestinal inflammation. Physical examination showed tachycardia and abdominal tenderness. Laboratory data were the following; hemoglobin (Hb) 12.0 g/dL (normal: 11.3-14.9 g/dL), white blood cell count 9000 /μL (4000–9500 /μL), platelet count 29.1 × 10^4^ /μL (11.0-40.0 × 10^4^ /μL), serum albumin 3.1 g/dL (4–5 g/dL), serum IgG 229 mg/dL (870–1700 mg/dL), serum IgA, <24 mg/dL (110–410 mg/dL), and serum IgM, <18.6 mg/dL (33–190 mg/dL). Colonoscopy revealed multiple longitudinal ulcers in the ileum end and the ascending colon (Figure 1a-b). Cobblestone change of the mucosa of the terminal ileum was also observed (Figure 1a). No abnormality was found in the rectum and anus. Pathology examination showed erosion, distortion of crypts, and infiltration of leukocytes, although granulomas were not found (Figure 2). Most of the inflammatory cells in the intestinal mucosa were cluster of differentiation (CD) 3+ T cells, whereas CD 20+ B cells were not observed (Figure 2). Cytomegalovirus was serologically and pathologically negative. Taken together, we concluded that these findings were consistent with Crohn’s disease. After admission, he was given mesalazine and intravenous hyperalimentation, which did not improve his symptoms. Oral administration of 50 mg prednisone was given with very limited success. A month after admission, he had a massive melena and his hemoglobin fell to 5.5 mg/dL. Colonoscopy demonstrated an exposed blood vessel in one of the ulcers in the ascending colon, which was considered to as a potential bleeding point (Figure 3a). Although hemostasis was temporarily achieved by colonoscopic clipping (Figure 3b), he repeatedly experienced melena. He underwent emergency colonoscopy and blood infusion several times in short period. Thus, after excluding the possibilities of active infectious diseases by repeated fecal cultures, chest-abdominal computed tomography, and tuberculosis tests, infliximab was introduced under careful monitoring; venous injection of 250 mg infliximab was given on week 0, week 2 and week 6. Gastrointestinal bleeding from the ulcer halted after the first infliximab injection. Remarkable improvement was seen after the third infliximab injection; he was in clinical remission and deep ulcers in the terminal ileum had healed. Only small erosions in the ascending colon were present on follow-up colonoscopy (Figure 4a-b). Pathological findings also showed improvement with reduced infiltration of CD3+ T cells (Figure 5). Given his history of tuberculosis and repeated infections due to underlying CVID, we decided to terminate the infliximab therapy. He was discharged without side effects and has been in clinical remission for 3 years.

## Discussion

Dysfunction of the regulatory systems maintaining the balance between active immunity and tolerance in the gastrointestinal tract can result in mucosal inflammation. Thus, not surprisingly, CVID displays a wide spectrum of patterns including infectious colitis, lymphocytic colitis, collagenous enteritis, celiac diseases, and IBD-like colitis [2,8-10]. These conditions may be induced via autoimmunity and altered immune response towards the bacterial flora, although the precise mechanisms are yet to be determined [5]. Several diseases with immunodeficiency including CVID are associated with Crohn’s disease-like intestinal inflammation; Resnick *et al.* reported that in 473 patients with CVID followed over 4 decades, 20 (4.2%) developed IBD-like colitis [11]. Distinguishing IBD from infectious disease, especially in CVID patients, can be challenging since there is a high prevalence of infections enterocolitis in these patients and they both can show the same symptoms [2]. Crohn’s-like disease is generally diagnosed years after the diagnosis of CVID, but in some cases, colitis can be found before underlying immunodeficiency is discovered [8]. As observed in classic Crohn’s disease, CVID-related colitis can present with diarrhea**,** abdominal pain**,** and fever with endoscopic findings of longitudinal ulcers and cobble stone appearance that preferentially occurs in proximity to the ileum end. Pathology tests can show infiltrating inflammatory cells including T cells and granulomas as well as lack of plasma cells in majority of the patients [8,12]. Previous reports suggest that lack of immunoglobulin production may not directly participate in pathogenesis of CVID-related enterocolitis; an evidence to support this theory is that immunoglobulin supplementation does not improve the gastrointestinal symptoms. Rather, abnormal cytokine production through a T-cell receptor-mediated pathway more likely one of the key players that contribute to mucosal inflammation [2,8]. In fact, T cell aggregates are observed in the mucosa of the small intestine in approximately half of CVID patients [8]. Interestingly, a subgroup of CVID even has increased production of tumor necrosis factor; a cytokine that is known to play a significant role in pathogenesis of IBD [13]. Nevertheless, heterogenic features of CVID suggest that multiple rather than singular pathways may be involved in intestinal inflammation [2], and it is difficult to completely rule out the possibility that our patient incidentally had genetic or environmental background that could cause classic Crohn’s disease.

It has been described that treatment of Crohn’s-like disease in CVID patients is similar to the patients with classic IBD, including corticosteroids and immunosuppressive drugs [6]. Use of corticosteroids has been reported to raise risk of infections whereas immunosuppressive reagents likely do not compromise immune function to a significant degree [2,3,6,14]. Treatment with anti-TNFα-antibodies has been previously reported in 3 cases, which are shown in Tables 1 and 2 together with the current case [3,6]. There is another case series of CVID-related colitis which were successfully treated by infliximab [14], but these cases were not included in the table due to lack of colonoscopic/histological characteristics of Crohn’s disease. All the patients were male in their 20s or 30s (Table 1). Three cases including ours had undergone steroid treatment with limited or no effect before anti-TNF alpha therapy (Table 1). Three cases were treated with infliximab, and one with adalibmab (Table 2). In all 4 patients, at least some improvement was observed after anti-TNF alpha therapy without significant side effects (Table 2). In the current case, infliximab was successful to control the intestinal bleeding in acute phase, suggesting that similar to classic Crohn’s disease, anti-TNF alpha therapy can also induce rapid effect to control severe symptoms in CVID-related colitis. Furthermore, we note that only 3 courses of infliximab injection were effective enough to maintain 3 years of remission. In classic Crohn’s disease, main safety problem with the infliximab administration is formation of antibodies to infliximab, leading to loss of response and infusion reactions (especially when injected after discontinuing for some period) [15]. However, these issues may be less of a concern in CVID patients because of their disability to produce antibody. Further accumulation of the cases is required to determine if it is beneficial to continue anti-TNF alpha antibodies after remission in these patients. Another question would be whether immunosuppressive reagents such as azathioprine should be added in these patients. In our case, azathioprine was not considered because infliximab *per se* was sufficient to control the colitis. In other 3 cases, azathioprine has been used together with anti-TNF alpha treatment (Table 2). Since CVID is a disease with variability, they may respond to the treatment differently thus each case may as well be evaluated for need of supplementary treatments in combination with anti-TNF alpha antibodies. Although previous reports as well as our case did not show significant infection due to anti-TNF alpha therapy in CVID patients (Table 2), they should be carefully monitored for fungal infections, and injection of immunoglobulin is crucial to avoid severe complications.

**Table 1:** Baseline characteristics of four patients with common variable immunodeficiency (CVID) complicating Crohn’s-like colitis treated with anti-TNF alpha antibodies

**Case#**	**Report**	**Age**	**Sex**	**Location of the lesion**	**Colonoscopy finding**	**Pathological findings**	**Previous steroid treatment (outcome)**
1	Nos et al. [3]	20	M	Ileum, rectum, anus	Ulcers	Not described	Yes (unsuccessful)
2	Nos et al. [3]	24	M	Ileum, colon	Not described	Not described	No
3	Vázquez-Morón et al. [6]	33	M	Ileum, colon	Shallow ulcers	Crypt. distortion leucocyte accumulation, depletion of plasma cells,	Yes (steroid dependency)
4	Current case	26	M	Ileum, colon	Deep ulcers, cobble stone-like appearance	Crypt. distortion infiltration of T lymphocytes depletion of plasma cells,	Yes (unsuccessful)

**Table 2:** Details and Outcomes of the patients treated with anti-TNF alpha antibodies

**Case#**	**Anti-TNF alpha**	Duration of anti-TNF* alpha therapy	**Combined azathoprine**	**Side effects**	**Response to anti-TNF alpha therapy**
1	Infliximab	1 year (discontinued)	Yes	No	Remission
2	Adalibmab	More than 1 year (discontinued)	Yes	No	Partial response
3	Infliximab	Several weeks (continued)	Yes	No	Remission
4	Infliximab	4 weeks (discontinued)	No	No	Remission

*TNF: tumor nerosis factor.

## Conclusion

In conclusion, we experienced a case of massive intestinal bleeding due to Crohn’s-like disease in CVID, which promptly responded to short course infliximab and lead to mucosal healing. To our knowledge, this is the first case to show that infliximab is a useful therapy that can manage this acute and life threatening complication of CVID-related enterocolitis.

## Consent

Written informed consent was obtained from the patient for publication of this Case Report and any accompanying images in agreement with the Helsinki Declaration. A copy of the written consent is available for review by the Editor-in-Chief of this journal.

## Abbreviations

CVID: Common variable immunodeficiency; Ig: Immunoglobulin; Hb: Hemoglobin; CD: Cluster of differentiation; TNF: Tumor Necrosis Factor; NSAIDs: Non-steroidal anti-inflammatory drugs; PPI: Proton pump inhibitor.

## Competing interests

The authors declare that they have no competing interests.

## Authors’ contributions

YA, HY, and FT carried out the studies and data analyses and drafted the manuscript. DI, TK, KM, HM, NY, KO, HI, MN, and NN participated in its design and coordination and helped to draft the manuscript. TH participated in pathological examination and critical review of the manuscript. All authors read and approved the final manuscript.
